# Expression of estrogen receptor beta and overall survival in non-small cell lung cancer patients: Protocol for a systematic review and meta-analysis of cohort studies

**DOI:** 10.1097/MD.0000000000017559

**Published:** 2019-10-25

**Authors:** Haisheng Chen, Mi Yan, Wenna Shi, Jing Shi, Cunxian Duan, Qing Fan, Yanhong Wang, Hui Li

**Affiliations:** a Department of Pharmacy, Shandong Cancer Hospital and Institute, Shandong First Medical University and Shandong Academy of Medical Sciences,; b Department of Pharmacy, The Second Hospital of Shandong University, Jinan, China.

**Keywords:** estrogen receptor beta, non-small cell lung cancer, overall survival

## Abstract

**Background:**

Lung cancer is the leading cause of cancer-related deaths among males and the second leading cause among females worldwide. Numerous studies have linked estrogen status to lung cancer outcome. However, there are studies with conflicting results about the effect of ERβ on survival of lung cancer. The aim of this meta-analysis is to evaluate the prognostic impact of estrogen receptor beta expression on survival among NSCLC patients.

**Methods:**

We will search 15 electronic databases, including PubMed, Web of Science, EMBASE, Cochrane Library, and CNKI from inception to June 1, 2019. We will include all cohort studies comparing overall survival of NSCLC patients with high or low estrogen receptor beta expression. The database searches will be supplemented by searching through citations and references. Two reviewers will independently screen search results to identify eligible articles, complete data collection, and conduct quality assessment. All disagreements will be resolved by an independent third reviewer. Methodological quality of the included studies will be assessed using the Newcastle- Ottawa scale. Discrepancies will be resolved by consensus or by consulting a third author. Meta-analyses will be performed, and findings will be reported according to the preferred reporting items for systematic reviews and meta-analyses (PRISMA) and the meta-analysis of observational studies in epidemiology (MOOSE) guidelines.

**Results:**

The results will be submitted to a peer-reviewed journal for publication.

**Conclusion:**

This review will provide a comprehensive evaluation of the evidence on the prognostic impact of ERβ expression among NSCLC patients and will help clinicians find potential treatments based on estrogen signaling.

## Abstract 2

Supplemental Digital Content is available in the text

## 1 Introduction

Lung cancer is the leading cause of cancer-related deaths among males and the second leading cause among females worldwide.^[1,2]^ Clinicians face many difficulties in the management of lung cancer patients. In addition to EGFR, ALK etc, the estrogen receptor is another promising targeting gene.^[3]^ Many studies have demonstrated that estrogens (ERS) and estrogen signaling play a significant role not only in normal lung development but also in lung cancer pathophysiology.^[4,5]^ Cell lines derived from lung tumors of both men and women express estrogen receptors (ER) and respond to estrogens. Estrogen can be synthesized in the lung by the enzyme aromatase (CYP19A1; ref. 18). Aromatase is present in NSCLC cells and tumor tissues and is functional. Estrogens are known to stimulate non–small cell lung cancer (NSCLC) cell proliferation, whereas the antiestrogen fulvestrant inhibits this effect.^[6]^ Cellular responses to estrogens are mediated by 2 distinct receptors, ERa and ERβ. In lung cancer cells, ERβ is sufficient to induce the full range of estrogenic responses when no detectable full-length ERa protein is present. ^[7]^ And ERβ appears to be the predominant form in lung cancer from the literature.^[8–10]^

Numerous studies have linked estrogen status to lung cancer outcome. ^[6,7,11–21]^ ERβ has been reported to adversely affect the prognosis of lung cancer patients.^[6,12–14,16,17,21]^ However, there are several studies with conflicting results about the effect of ERβ on survival of lung cancer.^[18–20]^ Based on the discordant results, we conducted this meta-analysis in order to evaluate the prognostic impact of ERβ expression and location on survival among NSCLC patients.

We understand our systematic review to be an extension and update to the meta-analysis by Zhuang Luo and the meta-analysis by Lihong Ma.^[22,23]^ LUO suggested ERβ was significant associated with good overall survival in NSCLC patients on univariate analysis, while Ma get an conclusion that ERβ overexpression indicates no relationship of prognosis. LUO published the meta-analysis in 2015 and included totally 14 studies, including 4 Chinese studies. Ma published the meta-analysis in 2016 and included totally 11 studies, excluded the 4 Chinese studies and added a Spain study. Until now, at least three new studies have been published.^[24–26]^

## 2 Methods

This is the second study of literature, so no ethical approval and patient consent is required. The protocol outlines our strategy to conduct a systematic review of cohort studies on the effect of ERβ expression on survival among NSCLC patients informed by the Preferred Reporting Items for Systematic Reviews and Meta-Analysis (PRISMA) and the Meta-analysis of Observational Studies in Epidemiology (MOOSE) statements.^[27,28]^ We will adopt the four-phase PRISMA flow diagram. This study protocol has been developed in line with the Preferred Reporting Items for Systematic Reviews and Meta-Analysis Protocols (PRISMA-P)^[27,29]^ statement and is registered with the International Prospective Register of Systematic Reviews (PROSPERO; registration number: CRD42019137949) This protocol will be updated as required by PRISMA-P criteria,^[29]^ and amended versions will be made available on PROSPERO.

### 2.1 Literature research

Extensive literature searches will be performed in the 15 databases PubMed, Web of Science, EMBASE, Cochrane Library, Cochrane Central Register of Controlled Trials (CENTRAL), Cochrane Database of Systematic Reviews (CDSR), CINAHL, Open- Grey, metaRegister of Controlled Trials, Clinical- Trials.gov, WHO Clinical Trials Database, WangFangData, CQVIP, COMPENDEX, and CNKI, from inception through June 1, 2019. Search terms include:

(1) “estrogen receptor”, “ER”, “ERβ” estrogen receptor beta
(2) non-small cell lung cancer or NSCLC or lung cancer or lung carcinoma or carcinoma of lung and
(3) outcome or survival or prognosis or OS.

Furthermore, we will hand search the citations and references of the identified literature for more potentially relevant studies to ensure literature saturation. Finally, we will conduct a grey literature search to consider unpublished studies (e.g., conference abstracts) using Google and Google Scholar with the search terms named above. If applicable and necessary, we will try to gather any other non-published data by contacting the researchers directly. We restrict publications to the languages English and Chinese.

### 2.2 Inclusion criteria and exclusion criteria

We will search for recent results from observational studies (study design criteria) conducted in NSCLC patients (setting criteria) reporting the OS (outcome criteria) in at least 2 different ERβ expression levels using comparable methods (design criteria). We will only include studies for which a detailed reporting of ERβ test and evaluation methods is available in order to be able to evaluate individual study quality and methodological differences between studies. We include primary non-small cell lung cancer patients and excluded patients with other cancers or serious diseases.

The exposure group is defined as a group of NSCLC patients who express high level of ERβ while the contrast group is defined as a group of NSCLC patients with low level of ERβ.

### 2.3 Selection of studies

Two reviewers, namely Haisheng Chen and Wenna Shi, will independently identify potentially eligible articles by screening all titles and abstracts of the hits from the databases. At this stage, articles will be classified as relevant, irrelevant or uncertain. Articles classified as irrelevant will be excluded and reasons for that decision will be given. For articles judged relevant or uncertain, full texts will be obtained. Then a full-text analysis will be undertaken, again independently by the 2 reviewers, to finally estimate study eligibility based on the previously established criteria. Any discrepancies in each stage of the study selection process will be resolved by discussion between the 2 reviewers. In cases of disagreement about particular articles, a third opinion from Cunxian Duan will be obtained.

### 2.4 Data collection process and data items

A standardized data abstraction form will be used to extract information on study characteristics, participant characteristics, relevant outcomes and methodology. A pilot version of the data extraction form (see Sheet 1, Supplemental Content, which will record Individual Characteristics and results of eligible prognostic studies evaluating survival) will be tested independently by 2 reviewers on a subsample of the included studies to ensure that all relevant information is covered. A discussion of the first extraction experience will follow and any emerging issues will be corrected in the finalized form.

Data from each study will be collected independently and in duplicate by Chen and Shi. Whether data abstraction is reliable will be tested on a random sample. If necessary, modifications may be made and, in cases of disagreement, a third researcher (Duan) will be involved. If any data cannot be clearly extracted, the study authors will be contacted.

In particular, we seek to extract the following variables:

(1) study characteristics (e.g., author, year of publication, country, study year(s), design, sample size(s),
(2) participants’ characteristics (e.g., age, gender),
(3) measurements (e.g., laboratory methodology, scoring system, cut-off value, HR with 95% CI) and
(4) methodological aspects (e.g., patients inclusion/exclusion criteria, study limitations).

### 2.5 Data management

References and data will be managed using the Review Manager (RevMan) software package, version 5.3 (by the Nordic Cochrane Centre, The Cochrane Collaboration, Copenhagen, Denmark, 2014. RevMan is specifically designed for managing and analyzing data in reviews applicable from bibliographical management to data synthesis.

### 2.6 Assessment of methodological quality

Two reviewers will independently score the quality of included studies using the Newcastle-Ottawa scale (NOS).^[30]^ The NOS assesses nonrandomized/observational studies based on 8 items categorized into 3 groups:

(1) the selection of the study groups,
(2) the comparability of the groups, and
(3) the ascertainment of either the exposure or outcome of interest.^[30]^

Discrepancies will be resolved by consensus or by consulting the third investigator (Duan). Agreement on screening, data abstraction, and methodological quality will be measured using the Kappa statistic.^[31]^

### 2.7 Data synthesis

We will both provide a narrative synthesis and, if appropriate, conduct a quantitative meta-analysis using funnel and forest plots, and pooled statistics. To do so, we will extract and pool outcome data from the selected studies to identify similarities and critical differences in clinical assessments, study design, and the methodological and statistical approach. We will use RevMan for implementing the characteristics of studies, preparing the review, and building the tables and plots.

#### 2.7.1 Assessment of heterogeneity

We will inspect and test for heterogeneity in study characteristics using forest plots and statistics such as χ2 tests (significance level a priori set at *P* < .1) and I2 values for pairwise meta-analysis. According to the Cochrane Handbook, we suppose a moderate level of heterogeneity between studies for I2 values ranging from 30% to 60%. If I2 exceeds 60% for the pooled analysis, we will explore sources of heterogeneity in subgroups of studies. Depending on the observed heterogeneity, we will decide to use fixed -effect, random-effect or mixed-effect models to estimate the overall survival and to quantify the uncertainty of that estimate.

#### 2.7.2 Sensitivity/subgroup analyses

Given that we will be able to include a sufficient number of studies, we will perform additional analyses in order to check the robustness of our analytical approach. If data are sufficiently available, we aim to repeat the meta-analysis including only studies of considerable quality (i.e., low risk of selection bias, low risk of nonresponse bias). Further, we will undertake subgroup analyses to investigate whether covariates exist and to examine heterogeneity in our outcome. Analyses will be performed for subgroups stratified by patient age and sex, ERβ location (cytoplasmic or nuclear expression), laboratory method, country, and study risk of bias (low versus high).

#### 2.7.3 Meta-bias

If a sufficient number of studies can be included in the meta-analysis, we will use graphical (e.g., funnel plots) and statistical (e.g., Egger tests) methods to explore the presence of small-study effects, which are indicative of possible publication bias.^[32,33]^

### 2.8 Confidence in cumulative evidence

We will assess the quality of the supporting evidence of each included study by using the GRADE methodology.^[34]^ This allows for the assignment of four grades of evidence (high, moderate, low, very low quality) to 5 different domains: limitations (risk of bias), imprecision, inconsistency, indirectness and publication bias. We will use the GRADEpro online software tool from the Cochrane collaboration (GRADEpro V.3.6; available from http://gradepro.org) to import results of statistical analyses from RevMan and export a summary of the findings table back into the RevMan file. We will provide a table in our systematic review summarizing the quality assessment of included publications.

## 3 Discussion

The systematic review and planned meta-analysis will provide a comprehensive overview of the evidence of the prognosis effect of ERβ expression in NSCLC. There have been conflicting results, and even 2 published meta-analyses have come to different conclusions. We aim to provide a critical discussion on the causes of discordant outcomes from different cohorts.

The results of the systematic review may be limited by study design. If the reviewed articles prove too heterogeneous, a meta-analysis may not be feasible, and hence, a summarizing statement of the evidence of prognosis value of ERβ in NSCLC would be impossible.

If there is further evidence of prognosis value of ERβ in NSCLC, this could have implications for clinicians. In particular, it would support the estrogen or the anti-estrogen drugs to treat NSCLC. This review will also identify any gaps in the current literature on this topic and provide direction for future research in this area of study.

Sheet 1, Supplemental Content, which will record Individual Characteristics and results of eligible prognostic studies evaluating survival

## Author contributions

HL and HC are the guarantors of the systematic review. WS and CD drafted the manuscript. All authors contributed to the conception and design of the review. JS, QF and YW developed the search strategy. HL and MY critically revised the protocol for important intellectual content. All authors approved the final version of the manuscript.

Hui Li orcid: 0000-0001-5583-5329.

## Supplementary Material

Supplemental Digital Content
